# The Work of the Panel Committees

**Published:** 1922-10

**Authors:** Alfred Cox

**Affiliations:** Medical Secretary, The British Medical Association (Medical Department), 429 Strand, W.C.2


### CORRESPONDENCE.

[Correspondence on all subjects is invited, but we cannot be responsible for the opinions expressed by our correspondents, who must give name and address as a guarantee of good faith, but not necessarily for publication. Correspondents are reminded that conciseness greatly facilitates early insertion.]

### The Work of the Panel Committees.

*To the Editor of* The Hospital and Health Review.

Sir,—I have read with great interest each of the articles on National Health Insurance as it affects the medical profession, contributed to your journal by Mr. R. W. Harris. The knowledge and impartiality shown by Mr. Harris are only, of course, what those of us who knew him well in his official position would expect, and I think you are doing a great service in publishing articles which, while giving fair play to the profession, have shown up some of the weak points in the National Health Insurance system.

The point I particularly wish to emphasise, however, is raised by Mr. Harris's appeal to the doctors concerned to take vigorously in hand cases of proved deficiency of surgery accommodation. I believe they will do so, and the reason why I believe this arises out of what I consider to be one of the best points in the National Health Insurance system, namely, that it lays upon the doctors themselves, through the Panel Committees, a great deal of the responsibility for the effective conduct of the service. With every alteration in the regulations, more and more responsibility has been thrown upon the Panel Committees. This I am sure is a good thing, because my experience is that, on the whole, representative insurance practitioners are far more critical of the *real* failings of their colleagues than any lay body. Panel Committees may be trusted to estimate at its true value the "swearing at large" so often indulged in by the smaller fry of the Approved Society world, but when it comes to real evidence that doctors under their control are guilty of sins either of commission or omission which are likely to bring the service into disrepute, I believe the public may safely rely upon the Panel Committees to do their duty fearlessly. I suppose no one has read more reports of the proceedings of Panel Committees than I have, and I have been much struck by the steadily increasing evidence that most of these Committees are taking their responsibilities very seriously. So I hope that the Ministry of Health will go on increasing the responsibility of the Panel Committees for the efficiency of the service rendered by their constituents, and that Insurance Committees, instead of trying to "crab" the panel doctors, as a few of them notoriously do, will make it evident that they look more and more to the Panel Committees to see that the medical service is worthy of the community which pays for it and uses it, and of the medical profession which provides it.

I sincerely hope you will continue Mr. Harris's articles, which I, for one, find both informative and stimulating.

Yours faithfully,

Alfred Cox,

Medical Secretary, The British Medical Association (Medical Department), 429 Strand, W.C.2

### Co-operation in a Contributory Scheme.

*To the Editor of* The Hospital and Health Review.

